# A Preliminary Genetic Analysis of Complement 3 Gene and Schizophrenia

**DOI:** 10.1371/journal.pone.0136372

**Published:** 2015-08-25

**Authors:** Jianliang Ni, Shuangfei Hu, Jiangtao Zhang, Wenxin Tang, Weihong Lu, Chen Zhang

**Affiliations:** 1 Tongde Hospital of Zhejiang Province, Zhejiang, China; 2 Zhejiang Provincial People’s Hospital, Zhejiang, China; 3 Hangzhou Seventh People’s Hospital, Zhejiang, China; 4 Schizophrenia Program, Shanghai Mental Health Center, Shanghai Jiao Tong University School of Medicine, Shanghai, China; Sudbury Regional Hospital, CANADA

## Abstract

Complement pathway activation was found to occur frequently in schizophrenia, and complement 3 (C3) plays a major role in this process. Previous studies have provided evidence for the possible role of C3 in the development of schizophrenia. In this study, we hypothesized that the gene encoding C3 (C3) may confer susceptibility to schizophrenia in Han Chinese. We analyzed 7 common single nucleotide polymorphisms (SNPs) of C3 in 647 schizophrenia patients and 687 healthy controls. Peripheral *C3* mRNA expression level was measured in 23 drug-naïve patients with schizophrenia and 24 controls. Two SNPs (rs1047286 and rs2250656) that deviated from Hardy-Weinberg equilibrium were excluded for further analysis. Among the remaining 5 SNPs, there was no significant difference in allele and genotype frequencies between the patient and control groups. Logistic regression analysis showed no significant SNP-gender interaction in either dominant model or recessive model. There was no significant difference in the level of peripheral *C3* expression between the drug-naïve schizophrenia patients and healthy controls. In conclusion, the results of this study do not support *C3* as a major genetic susceptibility factor in schizophrenia. Other factors in AP may have critical roles in schizophrenia and be worthy of further investigation.

## Introduction

Schizophrenia is a chronic, severe and disabling brain disorder that affects approximately 1% of worldwide population. In the past decades, schizophrenia has been regarded as a neurodevelopment disorder. Early literature reported that adverse conditions may result in abnormal brain development during the perinatal period, whilst schizophrenic symptoms appear in later life after the synaptic pruning process [1,2]. However, the pathophysiology of schizophrenia remains unknown [3].

Although heritability estimates for schizophrenia reach 80%, twin concordance is around 50% [4]. Hence, non-genetic factors also play an important role in this disorder [5]. It has been well-documented that maternal virus infection is one of the most consistently identified environmental risk factors for schizophrenia [6]. On the other hand, clinical observations indicated that schizophrenia and certain autoimmune diseases share some key clinical, epidemiological and genetic features [7]. Such findings suggested that immune abnormalities may be implicated with the pathophysiology of schizophrenia [8].

Complement acts as a rapid and efficient immune surveillance system that serves to protect the body against the invasion and proliferation of various microorganisms [9,10]. Complement pathway activation was reported to occur frequently in schizophrenia, in which complement 3 (C3) regulates the process [11]. C3 is a protein of the immune system that plays a central role in the complement cascade and contributes to innate immunity. Recent observations have demonstrated that C3 is a critical mediator for synaptic refinement and plasticity in neurodevelopment [12,13]. In comparison with healthy controls, Hakobyan et al. [14] observed a significant higher level of C3 protein in schizophrenia patients. As such, the above findings provide interesting clues for the potential role of C3 in schizophrenia.

At the molecular level, the gene encoding C3 (*C3*) is located at chromosome 19, which has been reported to be a genetic schizophrenia susceptibility region [15]. However, few genetic studies have been carried out to investigate the association of *C3* with schizophrenia and yielded inconsistent results [16,17,18]. It is known that *C3* contains 41 exons and spreads over 41kb. One weakness for the early genetic studies is too few polymorphisms tested. In the present study, we aimed to examine whether the region of *C3* is associated with schizophrenia. A total of 7 polymorphisms were selected for a better coverage of this region. As a secondary aim, prior study reported that *C3* has a gender-specific effect [19], which may underlie differential susceptibility to schizophrenia [20]. So we attempted to examine whether there was any gender difference in the association of *C3* with schizophrenia. Data has shown that *C3* polymorphisms result in alternations in its protein function. To validate previous findings, we opted to measure the serum *C3* expression level among drug-naïve schizophrenia patients and healthy controls.

## Methods

### Subjects

All subjects provided written informed consent prior to performing any of the procedures related to this study. All procedures were reviewed and approved by the ethical committees at Tongde Hospital of Zhejiang Province and Hangzhou Seventh People’s Hospital, and performed in strict accordance with the Declaration of Helsinki, and other relevant national and international regulations.

For the genetic analysis, a total of 647 schizophrenia patients recruited from Tongde Hospital of Zhejiang Province and The Seventh People’s Hospital of Hangzhou. The inclusion criteria for this study were according to our previous ones [20,21,22]. All patients (1) met the Diagnostic and Statistical Manual of Mental Disorders, Fourth Edition (DSM-IV) criteria for schizophrenia; (2) were not first-episode; (3) had no chronic physical disease or other psychiatric disorder aside from schizophrenia. Prior to analysis, all diagnosis and review of psychiatric case records were independently checked and verified by two senior psychiatrists. The control group comprised of 687 Han Chinese enrolled from the local community in Hangzhou. Before sampling, the volunteers self-reported that they were in good physical health and have no family history of psychiatric disorders. Those who have medical illnesses or drug and alcohol abuse/dependence were excluded. Demographic and clinical characteristics were presented in S1 Table.

For the expression analysis, twenty-three drug-naïve patients with first-episode schizophrenia were recruited from Tongde Hospital of Zhejiang Province. The patients were diagnosed according to the DSM-IV criteria for schizophrenia and had no physical disease. Twenty-four healthy subjects from Hangzhou city were also recruited for control group. Basic blood and urine tests were performed prior to recruitment in order to exclude any current physical illness. Patients and controls did not significantly differ for age, gender, BMI and smoking status. Detailed information was presented in S2 Table.

### SNP selection

We retrieved CHB data from the HapMap database (http://www.hapmap.org) and defined linkage disequilibrium (LD) blocks using Haploview 4.2 (Broad Institute, Cambridge, MA, USA) to set inclusion criteria for tagging SNPs. Haplotype-tagging single nucleotide polymorphisms (htSNPs) with *R* ^2^ cutoff>0.8 and minor allele frequency (MAF)>0.1 were selected. In total, three tag SNPs of *C3* were selected for genotyping. Four potential *C3* functional SNPs (rs7951, rs2230199, rs2250656 and rs11672613) [23,24,25,26] were also examined in this study (S3 Table).

### Genotyping

Genomic DNA of all participants was extracted from peripheral blood using a Tiangen DNA Isolation Kit (Tiangen Biotech, Beijing, China). All 7 SNPs were amplified independently via polymerase chain reaction (PCR) and then genotyped via direct sequencing on an ABI PRISM 3730 Genetic Analyzer (Perkin-Elmer Applied Biosystems). S4 Table detailed the primers information. Genotyping was carried out according to the methods described in our previous studies [27,28]. PCR amplification was performed in a volume of 25 μL containing primer pair for each SNP. PCR primers were also used for sequencing. Sequencing results were handled using DNAStar package (DNA Star Inc., USA), and the original sequencing chromatograms of each sample were then manually checked.

### Quantitative real-time polymerase chain reaction (qRT-PCR)

We carried out the *C3* mRNA expression analysis using qRT-PCR as previously described [29,30]. Peripheral blood was collected and mononuclear cells were separated by Ficoll-Paque PLUS density gradient centrifugation (GE Healthcare, Amersham, NJ, USA) within 2 hour, placed in TRIzol (Invitrogen, Carlsbad, CA, USA) and stored at -80°C. The total RNA was isolated from peripheral blood mononuclear cells according to the manufacturer’s protocol, and 2 μg total RNA was re-transcribed into complementary DNA with reverse transcription (ReverTra Ace, Toyobo, Osaka, Japan) according to the manufacturer’s instruction. Relative *C3* mRNA expression levels were assessed by real-time PCR with commercially available TaqMan gene expression assays for target gene *C3* and glyceraldehydes-3-phosphate dehydrogenase (*GAPDH*) as reference gene (Applied Biosystems, CA, USA). All experiments were conducted in optical 384-well reaction microtiter plates on an ABI Prism 7900HT Sequence Detection System (Applied Biosystems, CA, USA). PCR was performed in a total volume of 10μL containing 1×TaqMan Universal Master Mix with AmpErase UNG, 1×Assay Mix (Applied Biosystems, CA, USA) and complementary DNA template at cycle conditions: 95°C for 15 min, followed by 40 cycles at 95°C for 15s and 60°C for 60s. All reactions were run in triplicate. In each sample, the expression of *C3* was normalized to the expression of the reference gene. Results were reported in fold change using 2^-ΔΔCt^.

### Statistical analysis

The Hardy-Weinberg equilibrium testing and individual SNP association analyses were conducted using SHEsis (http://analysis.bio-x.cn). The odds ratio (OR) and corresponding 95% confidence interval (CI) were calculated with the major allele as reference. Pairwise linkage disequilibrium of all pairs of htSNPs was performed using HaploView 4.2 (Broad Institute, Cambridge, MA, USA), and the extent of linkage disequilibrium (LD) was measured by the standardized *D*’ and *R* ^2^. Referring to the previous report [19], logistic regression was performed with SNP-gender interaction to adjust the effect of gender on SNPs. For the expression analysis, ANCOVA was carried out with age, gender, smoking status and BMI as covariates controlled in the model, to minimize the potential effect of these factors on the expression levels of *C3* mRNA. The ANCOVA analysis was performed using SPSS 17.0 (SPSS Inc., Chicago, IL, USA). To adjust for multiple testing, the level of significance was corrected via Bonferroni correction. Power calculations were carried out using Quanto 1.2.3 (http://hydra.usc.edu/GxE).

## Results

**Fig 1:**
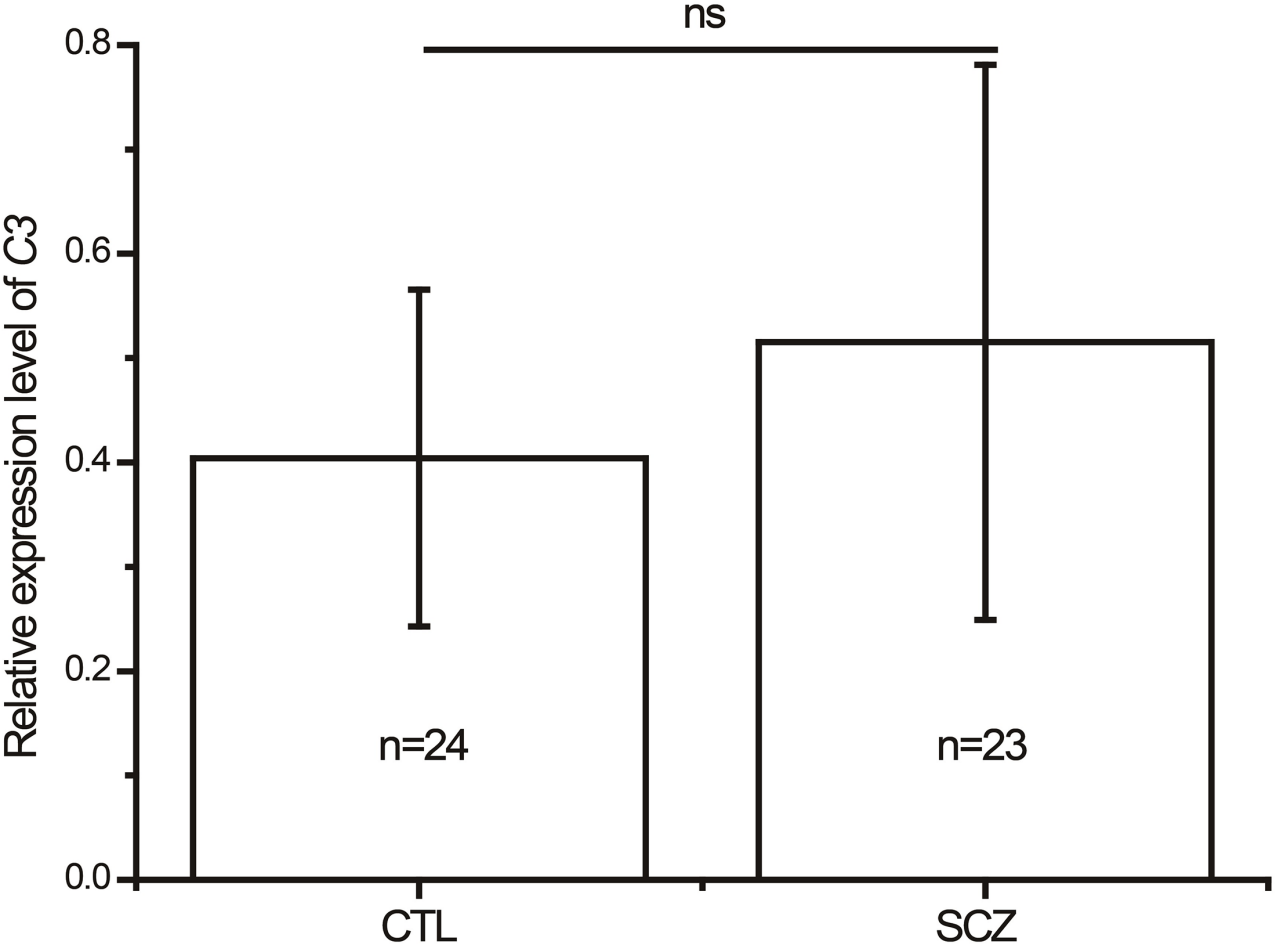
Expression levels of *C3* mRNA in peripheral blood in drug-naïve schizophrenia patients and healthy subjects. *C3* mRNA was normalized to that of *GAPDH*. CTL, control subjects (n = 24); SCZ, schizophrenia patients (n = 23); ns, no significance.

For the genetic analysis, there was no significant difference between the schizophrenia and control groups in term of age and gender. Seven SNPs were genotyped to investigate the association of *C3* with schizophrenia. Two SNPs (rs1047286 and rs2250656) that deviated from Hardy-Weinberg equilibrium were excluded for the further analysis. Among the remaining 5 SNPs, no deviation from the Hardy-Weinberg was observed in genotype distribution. Table 1 showed that there was no significant difference in allele and genotype frequencies between the patient and control groups. After calculating LD for all pairs of SNPs, we found a low *R* ^2^ in *C3* (S1 Fig), indicating that no specific haplotype block could be identified. We further investigate whether there was any gender difference in the association of *C3* with schizophrenia. Logistic regression analysis showed no significant SNP-gender interaction in either dominant model or recessive model (Tables 2 and 3). A total of 23 drug-naïve patients with schizophrenia and 24 well-matched healthy controls were recruited for the *C3* expression study. As shown in Fig 1, there was no significant difference in the level of peripheral *C3* mRNA expression between the drug-naïve schizophrenia patients and healthy controls. On the basis of the genotype data, the statistical power of the 5 SNPs within *C3* was more than 85% (*α* = 0.05) for schizophrenia samples under the assumption of a moderate effect size (OR = 1.5), a log additive model, and the prevalence of schizophrenia (≈1%).

**Table 1:** Comparison of genotype and allele frequencies of 5 *C3* SNPs in 647 schizophrenia patients and 687 healthy controls.

		Genotype, n (%)						Allele, n (%)				
rs2277984	N	G/G	G/A	A/A	*P* ^a^	*P* ^b^	N	G	A	OR (95%CI)	*P* ^a^	*P* ^c^
Case	647	132 (20.4)	336 (51.9)	179 (27.7)	0.95	0.26	1294	600 (46.4)	694 (53.6)	1.02 (0.88–1.19)	0.76	
Control	687	137 (19.9)	355 (51.7)	195 (28.4)		0.28	1374	629 (45.8)	745 (54.2)			
rs7951		T/T	T/C	C/C				T	C			
Case	647	7 (1.1)	122 (18.9)	518 (80.1)	0.42	0.95	1294	136 (10.5)	1158 (89.5)	0.91 (0.71–1.16)	0.45	
Control	687	5 (0.7)	147 (21.4)	535 (77.9)		0.13	1374	157 (11.4)	1217 (88.6)			
rs11672613		C/C	C/T	T/T				C	T			
Case	647	98 (15.1)	307 (47.4)	242 (37.4)	0.22	0.97	1294	503 (38.9)	791 (61.1)	0.90 (0.77–1.05)	0.17	
Control	687	109 (15.9)	352 (51.2)	226 (32.9)		0.15	1374	570 (41.5)	804 (58.5)			
rs2230205		A/A	A/G	G/G				A	G			
Case	647	141 (21.8)	346 (53.5)	160 (24.7)	0.09	0.07	1294	628 (48.5)	666 (51.5)	1.18 (1.01–1.37)	0.035	0.175
Control	687	127 (18.5)	357 (52.0)	203 (29.5)		0.17	1374	611 (44.5)	763 (55.5)			
rs2230199		G/G	G/C	C/C				G	C			
Case	647	0 (0.0)	10 (1.5)	637 (98.5)	0.50	0.84	1294	10 (0.8)	1284 (99.2)	0.76 (0.33–1.71)	0.50	
Control	687	0 (0.0)	14 (2.0)	673 (98.0)		0.79	1374	14 (1.0)	1360 (99.0)			

^a^ *P* values were not adjusted by Bonferroni correction

^b^ *P* values were calculated for Hardy-Weinberg equilibrium

^c^ *P* values were adjusted by Bonferroni correction.

**Table 2:** Logistic regression analysis of *C3* SNPs×gender interaction in dominant model.

Variables	B	S.E	Wals	OR (95%CI)	*P* ^a^	*P* ^b^
rs2277984×gender	0.54	0.22	5.87	1.71 (1.11–2.65)	0.015	0.075
rs7951×gender	-0.40	0.24	2.78	0.67 (0.42–1.07)	0.10	
rs11672613×gender	-0.23	0.21	1.26	0.79 (0.53–1.19)	0.26	
rs2230205×gender	0.21	0.21	0.95	1.23 (0.81–1.87)	0.33	
rs2230199×gender	-0.21	0.27	0.62	0.81 (0.47–1.38)	0.43	

^a^ *P* values were not adjusted by Bonferroni correction

^b^ *P* values were adjusted by Bonferroni correction.

**Table 3:** Logistic regression analysis of *C3* SNPs×gender interaction in recessive model.

Variables	B	S.E	Wals	OR (95%CI)	*P* ^a^
rs2277984×gender	0.04	0.24	0.02	1.04 (0.65–1.64)	0.88
rs7951×gender	0.35	1.25	0.08	1.42 (0.12–16.26)	0.78
rs11672613×gender	-0.17	0.26	0.43	0.84 (0.60–1.41)	0.79
rs2230205×gender	-0.14	0.23	0.37	0.87 (0.55–1.37)	0.55
rs2230199×gender	NA	NA	NA	NA	NA

^a^ *P* values were not adjusted by Bonferroni correction

NA, Not applicable.

## Discussion

As a key component of innate immunity, accumulating evidence has indicated that abnormalities in the complement system are implicated in the etiology of schizophrenia [23]. We have examined the association of schizophrenia with the gene encoding C4-binding protein (*C4BPB*/*C4BPA*), a potent circulating soluble inhibitor of the classical and lectin pathways of complement. However, our results did not support the involvement of *C4BPB*/*C4BPA* in schizophrenia [22]. Here, we aimed to investigate the association of *C3*, another critical factor in complement system, with schizophrenia in Chinese Han population.

In this study, we did not observe any significant difference of allele and genotype frequencies between the schizophrenia patients and healthy controls. The statistical power of our study was also enough to detect an association between the variants and schizophrenia. Although Liu et al. [19] reported a SNP-gender interaction in *C3*, we did not have such findings in our sample. These results demonstrated that there is no genetic association between *C3* and schizophrenia, at least in Han Chinese. However, a recent study showed that increased levels of C3, acting as activation of complement system, can be found in schizophrenia patients when compared with healthy controls [31]. In contrast, Wong et al. [32] found a lower level of C3 in schizophrenia patients than that in controls. We noticed that the patients in both studies were those with chronic schizophrenia [31,32]. The aforementioned inconsistent results prompted us to determine the expression of *C3* in drug-naïve patients with first-episode schizophrenia. Our results showed no significant difference in the level of *C3* expression between schizophrenia patients and healthy controls. Therefore, our findings suggested that *C3* may not confer susceptibility to schizophrenia in Han Chinese.

Recently, Li et al. [33] performed a label-free quantitative proteomics analysis to identify 27 proteins as being schizophrenia related proteins, and found dysregulation of the alternative complement pathway in schizophrenia patients. The alternative pathway (AP) is one of three complement pathways, which is initiated by the spontaneous hydrolysis of C3. A number of molecules are involved in the occurrence of AP. Even though no association of *C3* with schizophrenia was found in this study, we could not exclude possible role of AP in the development of schizophrenia.

On the other side, cytokines are believed to play a vital role in coordinating immunologic and inflammatory responses in physiological and pathological conditions [34]. Therefore, cytokines may be critical mediators of the cross-talk between immune system and neuropsychiatric disorders [35]. Miller et al. [36] meta-analyzed 40 studies on cytokines and schizophrenia, and observed significant alternations of cytokine network in schizophrenia. Therefore, imbalance of cytokine network may be involved in the pathophysiology of schizophrenia. Prior literature indicated that complement activation products, such as C3a and C3a desArg, may enhance cytokine synthesis and inhibit the systemic synthesis of proinflammatory cytokines [37]. It is known that schizophrenia results from the cumulative impact of multiple common small-effect genetic variants and interactions between genes with small effect may contribute a larger heritable proportion to the overall risk of this disorder [38]. Therefore, we assumed that interaction of *C3* with genes encoding cytokines may be more sensitive to account for its susceptibility to schizophrenia. There is a need for further investigations to validate this hypothesis.

This study has some limitations that should be noted. First, the lack of a significant association may be caused by the modest sample size, possibly resulting in a type II error. Second, we did not psychiatrically screen the control subjects. Third, the principal hypothesis underlying this study is that common SNPs within *C3* may confer susceptibility to schizophrenia. Therefore, we did not sequence the *C3* to assess the influence of rare variants on schizophrenia, and this prevented us to detect their active role in the development of this disorder. Fourth, the case-control association analyses have the potential for population stratification, although all participants were ethnically matched in our sample. Finally, Wong et al. [32] reported lower level of C3 protein in schizophrenia patients in comparison to healthy controls only in male subjects, suggesting that there might be interesting to test *C3* expression separately in male and female subjects. However, we recruited only 47 individuals with or without schizophrenia in the *C3* expression study. The small sample size limited us to further analyze the gender-specific effect of *C3* expression on schizophrenia. Meanwhile, this also limited us to detect the association of studied *C3* SNPs with *C3* mRNA.

In conclusion, the results of this study do not support *C3* as a major genetic susceptibility factor in schizophrenia. Other factors in AP may have critical roles in schizophrenia and be worthy of further investigation.

## Supporting Information

S1 Fig Linkage disequilibrium plots consisting of 5 SNPs within *C3*. (DOC)

S1 Table Demographics of schizophrenia cases and controls for genetic analysis. (DOC)

S2 Table Demographics of schizophrenia cases and controls for expression analysis. (DOC)

S3 Table Information of selected SNPs genotyped in this study. (DOC)

S4 Table Primers for genotyping the *C3* SNPs. (DOC)
