# Supplementary material for: A Preliminary Genetic Analysis of Complement 3 Gene and Schizophrenia
Source: PLoS One. 2015 Aug 25;10(8):e0136372. doi: 10.1371/journal.pone.0136372 (PMC4549269; doi:10.1371/journal.pone.0136372)
Supplement: S1 Fig — (DOC) [file pone.0136372.s001.doc]

S1 Fig. Linkage disequilibrium plots consisting of 5 SNPs within *C3*. Pairwise linkage disequilibrium (LD) was computed for all possible combinations using the values of D’ and R^2^.

**
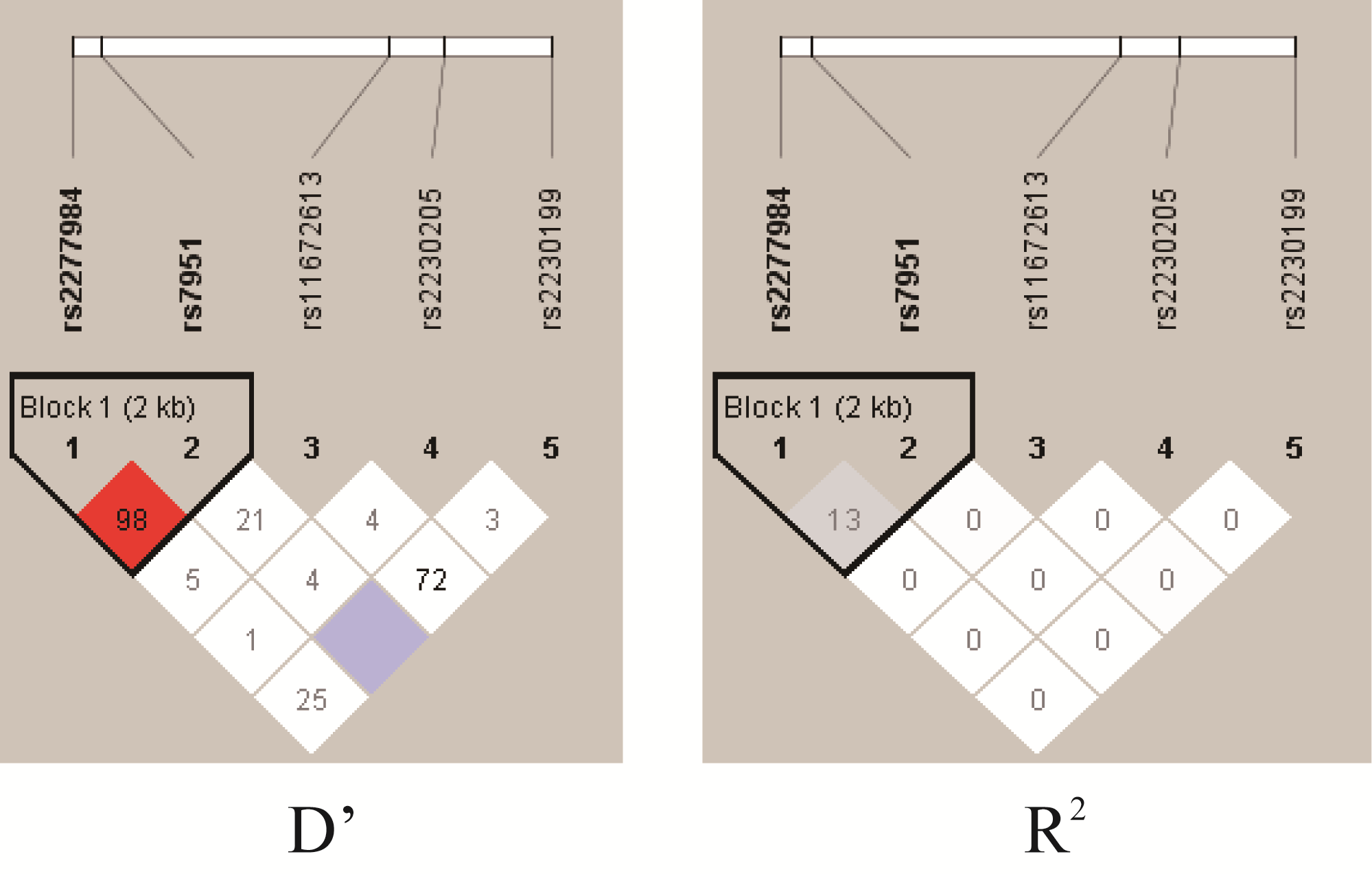
**
