# Supplementary material for: A Preliminary Genetic Analysis of Complement 3 Gene and Schizophrenia
Source: PLoS One. 2015 Aug 25;10(8):e0136372. doi: 10.1371/journal.pone.0136372 (PMC4549269; doi:10.1371/journal.pone.0136372)
Supplement: S1 Table — (DOC) [file pone.0136372.s002.doc]

S1 Table Demographics of schizophrenia cases and controls for genetic analysis

|  | Cases | Controls | *P* |
| --- | --- | --- | --- |
| Number of subjects (n) | 647 | 687 |  |
| Age (years), mean (SD) | 32.9 (6.7) | 35.1 (8.3) | 0.21 |
| Gender, male n (%) | 390 (60.3) | 413 (60.1) | 0.96 |
| Age at onset (years), mean (SD) | 27.4 (3.5) |  |  |
| PANSS total score, mean (SD) | 53.8 (16.9) |  |  |

PANSS, Positive and Negative Syndrome Scale
