# Supplementary material for: A Preliminary Genetic Analysis of Complement 3 Gene and Schizophrenia
Source: PLoS One. 2015 Aug 25;10(8):e0136372. doi: 10.1371/journal.pone.0136372 (PMC4549269; doi:10.1371/journal.pone.0136372)
Supplement: S2 Table — (DOC) [file pone.0136372.s003.doc]

S2 Table Demographics of schizophrenia cases and controls for expression analysis

|  | Cases | Controls | *P* |
| --- | --- | --- | --- |
| Number of subjects (n) | 23 | 24 |  |
| Age (years), mean (SD) | 30.3 (5.9) | 28.3 (6.1) | 0.25 |
| Gender, male n (%) | 15 (65.2) | 13 (54.1) | 0.56 |
| Smoking status, n (%) | 16 (69.6) | 11 (45.8) | 0.14 |
| BMI, mean (SD) | 24.2 (0.8) | 24.4 (0.7) | 0.50 |

Note: BMI, body mass index
