# Supplementary material for: A Preliminary Genetic Analysis of Complement 3 Gene and Schizophrenia
Source: PLoS One. 2015 Aug 25;10(8):e0136372. doi: 10.1371/journal.pone.0136372 (PMC4549269; doi:10.1371/journal.pone.0136372)
Supplement: S3 Table — (DOC) [file pone.0136372.s004.doc]

S3 Table Information of selected SNPs genotyped in this study

| SNP | Chromosome | Allele | Position | Function |
| --- | --- | --- | --- | --- |
| rs2277984 | 19 | G/A | 6630511 | intron |
| rs7951 | 19 | C/T | 6632991 | A1437A |
| rs11672613 | 19 | T/C | 6656246 | intron |
| rs2230205 | 19 | G/A | 6660704 | T612T |
| rs1047286 | 19 | C/T | 6664262 | P314L |
| rs2230199 | 19 | C/G | 6669387 | R102G |
| rs2250656 | 19 | G/A | 6669534 | intron |
