# Supplementary material for: A Preliminary Genetic Analysis of Complement 3 Gene and Schizophrenia
Source: PLoS One. 2015 Aug 25;10(8):e0136372. doi: 10.1371/journal.pone.0136372 (PMC4549269; doi:10.1371/journal.pone.0136372)
Supplement: S4 Table — (DOC) [file pone.0136372.s005.doc]

S4 Table Primers for genotyping the *C3* SNPs

| SNP | Allele | Product | Upper | Lower | Extend | SBE product |
| --- | --- | --- | --- | --- | --- | --- |
| rs2277984 | G/A | 90 | TTTGGAGGGAGGCCCTTA | TCCTCCTTTTCCGGATGG | TCTCATCTTCACATCACATCTGCCC | 26 |
| rs7951 | C/T | 141 | CAGGAGGGATTTTTCACAGG | ATGATGCAGCCTTACCTTGT | TCTCCAAGTATGAGCTGGACAAAGC | 40 |
| rs11672613 | T/C | 100 | AAAAGTACAGCCCACCATCC | ACAAAGAGGCGATAGTGTGAG | AACCATATTTCAGTAGACCAGACTT | 54 |
| rs2230205 | G/A | 102 | TGGTACTGGTGGCCGTGG | CTCTTCTCAGCAGCCTTGG | TGCTGAATAAGAAGAACAAACTGAC | 35 |
| rs1047286 | C/T | 90 | AGGTTGTGCTGAGCCGGA | TGGCAGACACGTACAAAGACT | GTACTGCTGGACGGGGTGCAGAACC | 30 |
| rs2230199 | C/G | 89 | CACGCCTAGATCCCAGCC | ACTTGGGTCCCGAAGGTG | CAGGGAGTTCAAGTCAGAAAAGGGG | 45 |
| rs2250656 | G/A | 92 | TCGCACCTCCTTCACATG | ATCTTGGGCTGGGTCCCT | TCCCTCCCCAAAACGGCCACCTCGG | 50 |
